# Recombinant CC16 inhibits NLRP3/caspase-1-induced pyroptosis through p38 MAPK and ERK signaling pathways in the brain of a neonatal rat model with sepsis

**DOI:** 10.1186/s12974-019-1651-9

**Published:** 2019-11-27

**Authors:** Ruixi Zhou, Xiaoyan Yang, Xihong Li, Yi Qu, Qun Huang, Xuemei Sun, Dezhi Mu

**Affiliations:** 1 Department of Pediatrics, West China Second University Hospital, Sichuan University, Chengdu, 610041 China; 2 Key Laboratory of Birth Defects and Related Diseases of Women and Children (Sichuan University), Ministry of Education, Chengdu, 610041 China

**Keywords:** Brain injury, ERK, p38 MAPK, Pyroptosis, Recombinant club cell protein (rCC16), Sepsis

## Abstract

**Background:**

Sepsis is a critical disease associated with extremely high mortality. Some severe forms of sepsis can induce brain injury, thus causing behavioral and cognitive dysfunction. Pyroptosis is a type of cell death that differs from apoptosis and plays an important role in the occurrence and development of infectious diseases, nervous system-related diseases. A recent study has found that there is pyroptosis in the hippocampus of sepsis-induced brain injury, but its mechanism and treatment scheme have not been evaluated.

**Methods:**

We established immediately a septic rat model by cecal ligation and perforation (CLP) after administration with recombinant club cell protein (rCC16) and/or U46619 in different groups. The clinical performance, survival percentage, vital signs, and neurobehavioral scores were monitored at different time points. Cortical pathological changes were also examined. The expression of cortical nucleotide-binding domain leucine-rich repeat-containing pyrin domain-containing 3 (NLRP3), caspase-1, (p)-p38 mitogen-activated protein kinase (MAPK), and (p)-extracellular signal-related kinase (ERK) was detected by western blotting and immunofluorescence analysis. The levels of interleukin (IL)-1β, IL-6, and tumor necrosis factor alpha in the cortical supernatant were detected by enzyme-linked immunosorbent assay.

**Results:**

Compared with the sham group, the clinical performance, survival percentage, vital signs, and severe cortical pathological changes in the CLP group were worse; NLRP3, caspase-1, and inflammatory factor levels were increased; and phosphorylation of p38 MAPK and ERK was also increased. Meanwhile, multiple indicators were deteriorated further after administration of U46619 in CLP rats. The clinical performance of CLP rats, however, was better after rCC16 administration; cortical pathological changes were attenuated; and NLRP3, caspase-1, and inflammatory factor levels and the phosphorylation of signaling pathway proteins (p38 MAPK and ERK) were reduced. Interestingly, the CLP rats showed the opposite changes in all indicators after administration with both rCC16 and U46619 when compared with those administered rCC16 alone.

**Conclusions:**

In sepsis, rCC16 inhibits cortical pyroptosis through p38 MAPK and ERK signaling pathways. Meanwhile, rCC16 has a protective effect on newborn rats with sepsis, but it is not clear whether its mechanism is directly related to pyroptosis.

## Background

Sepsis is an acute systemic inflammatory response caused by bacterial toxins, with high clinical mortality and the possibility of multiple organ damage and failure [1, 2]. When sepsis induces brain injury, it can lead to severe cognitive impairment and poor prognosis [3]. Severity of sepsis-induced brain injury is associated with multiple factors, such as inflammatory mediators, endotoxins, the dysfunction of the blood–brain barrier, and cell apoptosis [4, 5].

Pyroptosis, caspase-1-dependent cell death, is a recently identified pathway of host cell death closely related to inflammation. Under the action of bacteria, viruses, toxic foreign substances, etc., the caspase-1 is activated by nucleotide-binding domain leucine-rich repeat-containing (NLR), causing rapid cell membrane lysis and releasing intracellular pro-inflammatory factor, resulting in pyroptosis [6, 7]. Studies have shown that NLR pyrin domain-containing 3 (NLRP3)/caspase-1-mediated pyroptosis can lead to the maturation of inflammatory cells and cognitive dysfunction in mice with sepsis-related encephalopathy [5].

Club cell protein (CC16), a secretory protein found at extremely high levels in the lungs, is widely believed to be of great significance in anti-inflammatory and antioxidant stress [8, 9]. Studies performed to understand the roles of CC16 have revealed that recombinant CC16 (rCC16) not only shows therapeutic effects in disease models but also helps mitigate the respiratory function of premature infants and newborn lambs [10–12]. However, the role of rCC16 in the brain has not been reported.

Mitogen-activated protein kinases (MAPKs) are important components of signaling pathways in cells, which present receptor signals on the cell surface to the DNA in the nucleus. The p38 MAPK and extracellular signal-related kinase (ERK) are two important subgroups of MAPKs [13]. U46619 (9,11-dideoxy-9α,11α-methanoepoxy- prostaglandin F2α), an analogue of thromboxane A2, can promote platelet aggregation in vivo and in vitro. It has been reported that U46619 can further activate the p38 mitogen-activate protein kinase (MAPK) and extracellular signal-regulated kinase (ERK) signaling pathways by specifically binding to the thromboxane receptor [14–18]. Recent studies have shown that blocking the p38 MAPK signaling pathway can inhibit the pyroptosis of macrophages and reduce the release of inflammatory factors in acute lung injury [19]. Similarly, in macrophages, rCC16 can reduce tumor necrosis factor alpha (TNF-α), interleukin (IL)-6, and IL-8 release by inhibiting the p38 MAPK signaling pathway [20].

Considering the above, we aimed to verify whether (i) pyroptosis occurs in the cortex of newborn septic rats during brain injury, (ii) rCC16 can inhibit the activation of pyroptosis, and (iii) this process is mediated by the p38 MAPK and ERK signaling pathways. Finally, we hoped to shed light on the possibility of finding a feasible solution for the treatment of sepsis.

## Methods

### Animals and treatments

All experiments were conducted according to the guidelines of the Research Animal Care Committee of Sichuan University, and every effort was made to minimize the number and suffering of animals used. To avoid the influence of hormones on the experiment, 10-day-old male Sprague-Dawley rats (20–22 g, *n* = 324) were purchased from Sichuan Jianyang Dashuo Animal Science and Technology Co., Ltd. (Sichuan, China). All rats had free access to food and water and were maintained in an environment of 22–25 °C and 55–58% relative humidity on a 12-h light/dark cycle before and after cecal ligation and perforation (CLP).

The rats were randomly divided into the sham group, CLP group, CLP + rCC16 group, CLP + U46619 group, and CLP + rCC16 + U46619 group, with 36 rats in each group. In the CLP group, CLP + rCC16 group, CLP + U46619 group, and CLP + rCC16 + U46619 group, the rat model of moderate sepsis was established by CLP. After deep anesthesia, the abdominal cavity of the rats was opened along the midline of the abdomen, the cecum was ligated and punctured twice with a hollow needle, an appropriate amount of excreta was squeezed out, the cecum was then placed back into the abdominal cavity, and the wound was finally sutured layer-by-layer. However, in the sham group, after the abdominal cavity was opened, the cecum was turned over and then replaced, and then the abdomen was closed [21]. After CLP, rats in the sham group and CLP group were immediately injected with 5 μL 0.9% (w/v) saline into the lateral ventricle; rats in the CLP + rCC16 group were injected with 0.25 mg/kg rCC16 (PR018774, Sangon Biotech, Shanghai, China, soluble in 0.9% (w/v) saline, the dose was verified in previous experiments) at the same volume; rats in the CLP + U46619 group were injected with 10 mM U46619 (Santa Cruz, Dallas, TX, USA, soluble in 0.9% (w/v) saline) at the same volume); and rats in the CLP + rCC16 + U46619 group were injected with the same doses of rCC16 and U46619 [22, 23]. After drug administration, all rats were injected with 1 mL normal saline to prevent shock. The rats were sacrificed at the appropriate times and the samples were retained.

### Evaluation of vital parameters and survival percentage

First, the indwelling tube was placed in the femoral artery of the rats to connect the biological signal recorder (iWorx Systems, Dover, NH, USA) for monitoring of the dynamic changes in mean arterial pressure (MAP) and heart rate (HR). Next, the rats were placed in a suitable living environment with free access to food and water. We observed the mental and motor status, wound healing, and infection in the rats. The survival number of rats in each group was recorded over a specified time period.

### Neurological assessment

Neurobehavioral scoring was performed on rats to observe their neural reflexes to confirm the development of sepsis-induced encephalopathy. According to a previous study [24], a score of “0” indicated no reflex, “1” indicated reduced reflexes, and “2” indicated normal reflexes. A lower score was associated with more severe neurological damage to the brain. The pinna reflex, corneal reflex, righting reflex, tail flexion, and escape response reflex were evaluated.

### Histological examination

After deep anesthesia, the rats were injected with 100 mL 0.9% (w/v) saline and 100 mL 4% (w/v) paraformaldehyde (PFA) through the left ventricle for cardiac perfusion. The brain tissue was quickly removed and fixed in 4% PFA at 4 °C for 24–48 h. In the coronal plane, the brain tissue was trimmed, and only the part from the optic chiasma to the posterior with an additional 1 cm was retained. The prepared brain tissue was embedded in paraffin, cut into 6-μm sections, and stained with hematoxylin and eosin. Pathological changes in the tissue sections were observed, and images were captured under an optical microscope (Leica, Wetzlar, Germany).

### Western blot analysis

After the rats were deeply anesthetized at a specific time point, the entire brain was quickly removed, and the cortical tissue was isolated (from the optic chiasma to the posterior portion with 1 cm on the coronal plane) on ice. Each sample was placed in ice-cold lysis buffer (50 mM Tris-HCl pH 7.4, 150 mM NaCl, 10 mg/L NP-40, 0.1% protease inhibitor cocktail (Roche, Basel, Switzerland)) for 30 min. The sample was homogenized on ice and centrifuged 12,000×*g* for 30 min at 4 °C. The supernatant of the tissue was mixed with protein loading buffer (Beyotime, Shanghai, China) in proportion and denatured at 100 °C. A BCA analysis kit (Beyotime) was used to determine the protein content in the processed samples and ensure that the protein content in each sample was consistent. Each sample (20-μg brain tissue) was electrophoresed on a SDS-polyacrylamide gel with a density of 8% or 12%. The target proteins isolated from the gels were transferred to methanol-excited polyvinylidene difluoride membranes (Millipore, Billerica, MA, USA). The membranes were blocked in 5% milk prepared with Tris-buffered saline containing Tween 20 (TBS-T) for 1 h at room temperature. For immunoblotting, the membranes were incubated with primary antibodies with shaking overnight at 4 °C. The following primary antibodies were used: goat anti-NLRP3 polyclonal antibody (ab4207, 1:200, Abcam, Cambridge, UK), rabbit anti-caspase-1 polyclonal antibody (ab1872, 1:1000, Abcam), rabbit anti-phospho-ERK1/2 monoclonal antibody (8544, 1:1000, Cell Signaling Technology, Danvers, MA, USA), rabbit anti-ERK1/2 monoclonal antibody (4695, 1:1000, Cell Signaling Technology), rabbit anti-p38 (phospho T180 + Y182 polyclonal antibody (ab4822, 1:1000, Abcam), mouse anti-p38 monoclonal antibody (ab31828, 1:1000, Abcam), and mouse anti-tubulin monoclonal antibody (86298, 1:5000, Cell Signaling Technology). On the next day, the membranes were washed with TBS-T three times, and the corresponding secondary antibodies were incubated with the membranes. These secondary antibodies were as follows: horseradish peroxidase (HRP)-conjugated anti-goat secondary antibody (ab205723, 1:2000, Abcam), HRP-conjugated anti-rabbit secondary antibody (ZDR-5306, 1:5000, ZSGB-BIO, Beijing, China), and HRP-conjugated anti-mouse secondary antibody (ZDR-5307, 1:5000, ZSGB-BIO). The membranes were washed with TBS-T three times. Bands were visualized by enhanced chemiluminescence (Millipore) and imaged within the linear range by using a gel imaging analysis system (Bio-Rad, Hercules, CA, USA). In addition, the intensity of each band was quantitatively analyzed using Image J Analyzer Software (NIH, Bethesda, MD, USA) and the density ratio represented the relative intensity of each band against tubulin (as internal controls).

### Immunofluorescence staining

At specific time points, after deep anesthesia, the rats were injected with 100 mL 0.9% (w/v) saline and 100 mL 4% (w/v) PFA through the left ventricle for cardiac perfusion. The rats were immediately sacrificed, and their entire brains were removed. After trimming the excess brain tissue, only the coronal plane, optic chiasma, and an additional 1 cm remained; this sample was then fixed in 4% (w/v) PFA for 24–48 h at 4 °C. The immobilized brain tissue was then embedded in 2.5% (w/v) agarose and cut into 40-μm sections using an oscillating microtome (Leica). The sections were washed with phosphate-buffered saline (PBS) three times and then blocked with 0.3% (w/v) Triton X-100 for 30 min and blocking solution (containing 3% (w/v) bovine serum albumin (ST023, Beyotime), 2% (w/v) fresh bovine serum, and 0.2% (w/v) Triton X-100) for 1 h at room temperature. Primary antibodies were added, and the sections were incubated overnight at 4 °C. The goat anti-NLRP3 polyclonal antibody (1:50) and rabbit anti-caspase-1 polyclonal antibody (1:200) were used as primary antibodies. The sections were washed with PBS three times on the next day, and then incubated with secondary antibodies at room temperature and in the dark for 2 h. The secondary antibodies included DyLight 488-conjugated donkey anti-goat IgG (ab150129, 1:500, Abcam) and Cy3-conjugated donkey anti-rabbit IgG (711-165-152, 1:500, Jackson ImmunoResearch, West Grove, PA, USA). The sections were washed with PBS three times and then 4, 6-diamidino-2-phenylindole (C1002, DAPI, Beyotime) nuclear stain was applied (1:500 in PBS) in the dark for 10 min at room temperature. After staining, the sections were washed with PBS and sealed on the glass slide with anti-fluorescence quenching agent (Beyotime), followed by storage at 4 °C in a dark room. The sections were observed under a confocal laser scanning microscope (Olympus, Tokyo, Japan), and FV10-ASW-4.2 software (Olympus) was used to obtain images.

### Enzyme-linked immunosorbent assay

At specific time points after deep anesthesia, the rats were injected with 100 mL 0.9% (w/v) saline through the left ventricle for cardiac perfusion. The cortical parts (from the optic chiasma to the posterior portion of 1 cm on the coronal plane) of the brain tissue were isolated and stored at − 80 °C for later use. An interleukin (IL)-1β rat Enzyme-linked immunosorbent assay (ELISA) kit (ab100768, Abcam), IL-6 rat ELISA kit (ab100772, Abcam), and tumor necrosis factor (TNF) alpha rat ELISA kit (ab46070, Abcam) were used to detect these proteins. Samples were processed according to the manufacturer’s instructions. Absorbance was measured at different wavelengths with a microplate reader (Thermo Fisher Scientific, Waltham, MA, USA), and the final results were obtained according to different calculation formulas.

### Statistical analysis

The values used in the experimental results are expressed as the mean ± SEM, and SPSS version 19.0 software (SPSS, Inc., Chicago, IL, USA) was used for statistical analysis. Here, the statistical significance of the survival percentage of rats was analyzed by Kaplan-Meier method, whereas the statistical significance of differences between groups was analyzed by one-way analysis of variance followed by the Student-Newman-Keuls test. *P* < 0.05 was considered as statistically significant.

## Results

### Clinical performance and survival percentage

After modeling, the mental state and motor function of rats in the sham group were normal. Rats in the CLP group showed a delay in waking up after anesthesia. After waking up, the rats were listless; their bodies were curled up, and they were not willing to move and gather together to keep warm. They no longer took the initiative to eat or drink and their breathing rate was accelerated. Over time, the skin temperature of most rats decreased, muscle strength decreased, bloody secretions appeared around the eyes, and feces were loose and had a foul odor. In severe cases, shortness of breath occurred, following by death. In CLP + rCC16 group, the clinical performance of the rats was better than that in the CLP group, which was mainly reflected by increased activity and food and water intake, slower breathing, increased skin temperature, and no purulent secretions on the body surface. The clinical performance of the CLP + U46619 group was worse than that of the CLP group. Furthermore, compared with the CLP + rCC16 group, the clinical performance of rats in the CLP + rCC16 + U46619 group deteriorated, with the rats showing symptoms such as shortness of breath, decreased activity, body curling, decreased skin temperature, loose feces, and blood secretion around the eyes.

**Fig. 1:**
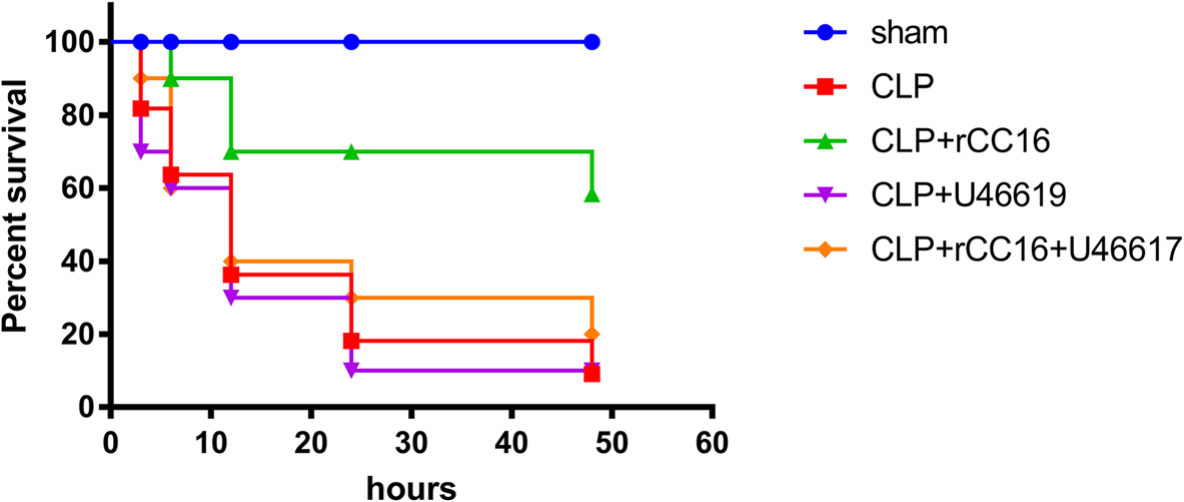
Survival percentage in each group at different time points. Survival percentage is represented by Kaplan-Meier curves. Values are expressed as mean ± SEM (*n* = 10 in each group)

As shown in Fig. 1, rats in the CLP group began to die at 3 h, with the highest mortality occurring between 6 and 12 h. The survival percentage at the end of 12 h was only 30% and that at 48 h was as low as 10% (*P* < 0.05). At 12 h, survival of the CLP + rCC16 group was 70%, which was significantly higher than that of the CLP group (*P* < 0.05). Survival of the CLP + rCC16 + U46619 group was 40% lower than that of the CLP + rCC16 group (*P* < 0.05). The CLP + U46619 group showed the same survival rate as the CLP group.

### Vital signs and neurobehavioral scores

**Fig. 2:**
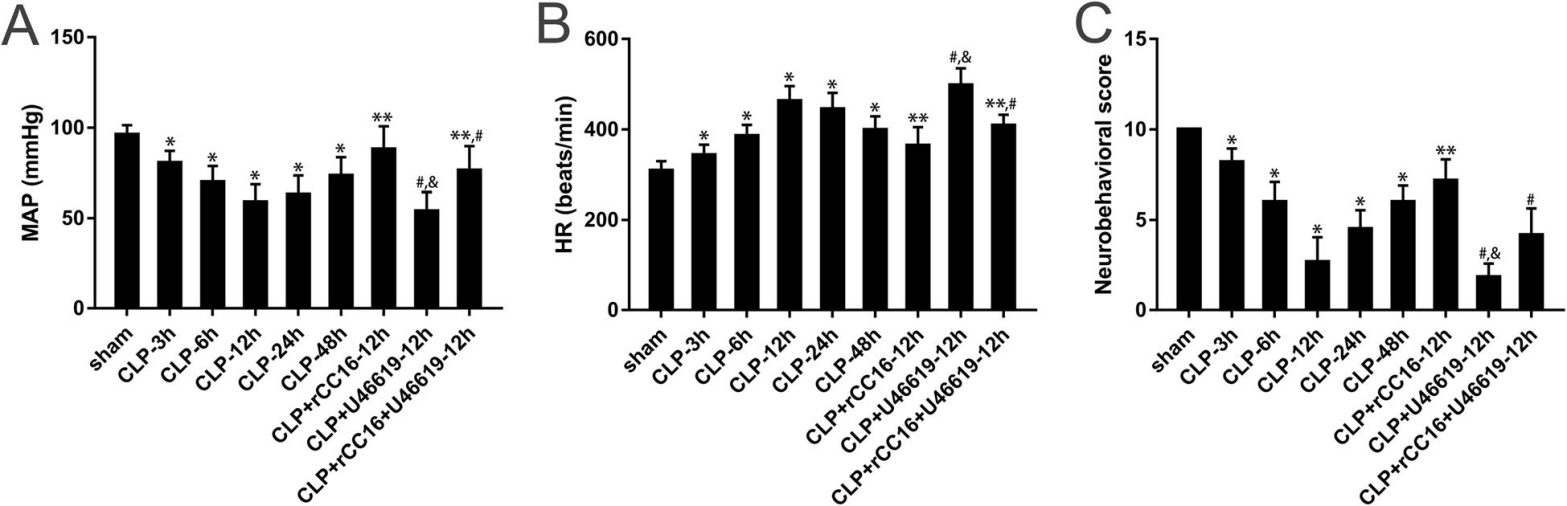
Mean arterial pressure, heart rate, and neurobehavior in each group at different time points. **a** Mean arterial pressure (MAP). **b** Heart rate (HR). **c** Neurobehavioral score. Data is represented by histograms. Values are expressed as mean ± SEM (*n* = 6 in each group; *P < 0.05 vs. sham group, **P < 0.05 vs. CLP-12 h, ^#^*P* < 0.05 vs. CLP + rCC16-12 h, ^&^*P* < 0.05 vs. CLP + rCC16 + U46619-12 h)

To determine the vital signs of the rats after CLP, we dynamically monitored the MAP and HR. After CLP, the MAP decreased gradually and HR increased when compared with in the sham group (96.2 mmHg, 309 beats/min); in the CLP-12 h group (59.0 mmHg, 463 beats/min), the MAP reached a minimum value, whereas HR showed a maximum value. Compared with the CLP group, the MAP of the CLP + rCC16 group (88.0 mmHg, 364 beats/min) increased significantly, while HR decreased; compared with the CLP + rCC16 group, the MAP of the CLP + rCC16 + U46619 group (76.3 mmHg, 409 beats/min) decreased, while HR increased. The MAP of the CLP + U46619 group (53.9 mmHg, 498 beats/min) was the lowest and HR was the highest in each group (*P* < 0.05, Fig. 2a and b).

To further investigate the effect of sepsis induced by CLP modeling on the brain, we evaluated the neurobehavior of the rats. The neurobehavioral score gradually reduced after awakening, with the lowest (2.67) observed at 12 h after surgical procedure in the CLP group, compared with the normal score (10) in the sham group. Additionally, compared with the CLP group, the score of the CLP + rCC16 group (7.17) was significantly improved. The scores for the CLP + rCC16 + U46619 group (4.17) and CLP + U46619 group (1.83) were both lower than those of the CLP + rCC16 group, with the CLP + U46619 group showing the lowest score (*P* < 0.05, Fig. 2c).

### Pathological changes

Sections were stained with hematoxylin and eosin to observe the pathological changes of cortex. The nuclei of healthy neurons were basophilic with clear nuclear boundaries, and cytoplasm and proximal axons were slightly eosinophilic, and the cell morphology was plumpness. In contrast, degenerating neurons were characterized by eosinophilic staining of both cell body and proximal dendrites; the intracellular structure was disordered [25]. The necrotic nerve cells were morphologically atrophy, vacuolation, karyopyknosis, and intact extracellular membrane [26].

**Fig. 3:**
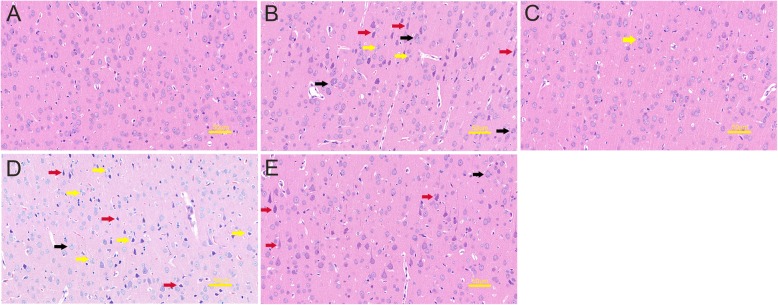
Pathological changes of the cortex detected in each group at 12 h. **a** Sham group, the cerebral cortex of the rats was normal. **b** CLP group, neuron degeneration (red arrows), nerve cell atrophy (yellow arrows), inflammatory cell infiltration (black arrows). **c** CLP + rCC16 group, the cortex was relatively normal, with occasional atrophy of nerve cells (yellow arrow). **d** CLP + U46619 group, brain tissue edema, a large number of degenerate neurons (red arrows), nerve cell atrophy (yellow arrows), visible inflammatory cell infiltration (black arrow). **e** CLP + rCC16 + U46619 group, some degenerative neurons (red arrows) and a small amount of inflammatory cell infiltration (black arrow) are seen. Scale bar = 50 μm

In the sham group, cortical cells were abundant, well-arranged, and normal in shape, and interstitial capillaries were abundant (Fig. 3a). The CLP group showed acute traumatic changes in the brain, which were most conspicuous at 12 h. In the cortex, some neurons were obviously degenerated (red arrows), some nerve cells atrophy and were slightly vacuolated (yellow arrows), and inflammatory cells had infiltrated the region (black arrows) (Fig. 3b). In the CLP + rCC16 group, the cortical pathological changes were significantly attenuated, and only a few nerve cells were atrophied (yellow arrow) (Fig. 3c). In the CLP + U46619 group, the pathological changes in cortex were the most severe, with loose brain parenchyma, pale staining of the tissues, overall degeneration of neurons (red arrows), vacuolation of nerve cells (yellow arrows), and inflammatory cell infiltration (black arrow) (Fig. 3d). In rats administered both rCC16 and U46619, degeneration of neurons (red arrows) and a small amount of inflammatory cell infiltration (black arrow) were observed in the cortex (Fig. 3e).

### Increase in NLRP3 and caspase-1 expression in the cortex after CLP surgery

**Fig. 4:**
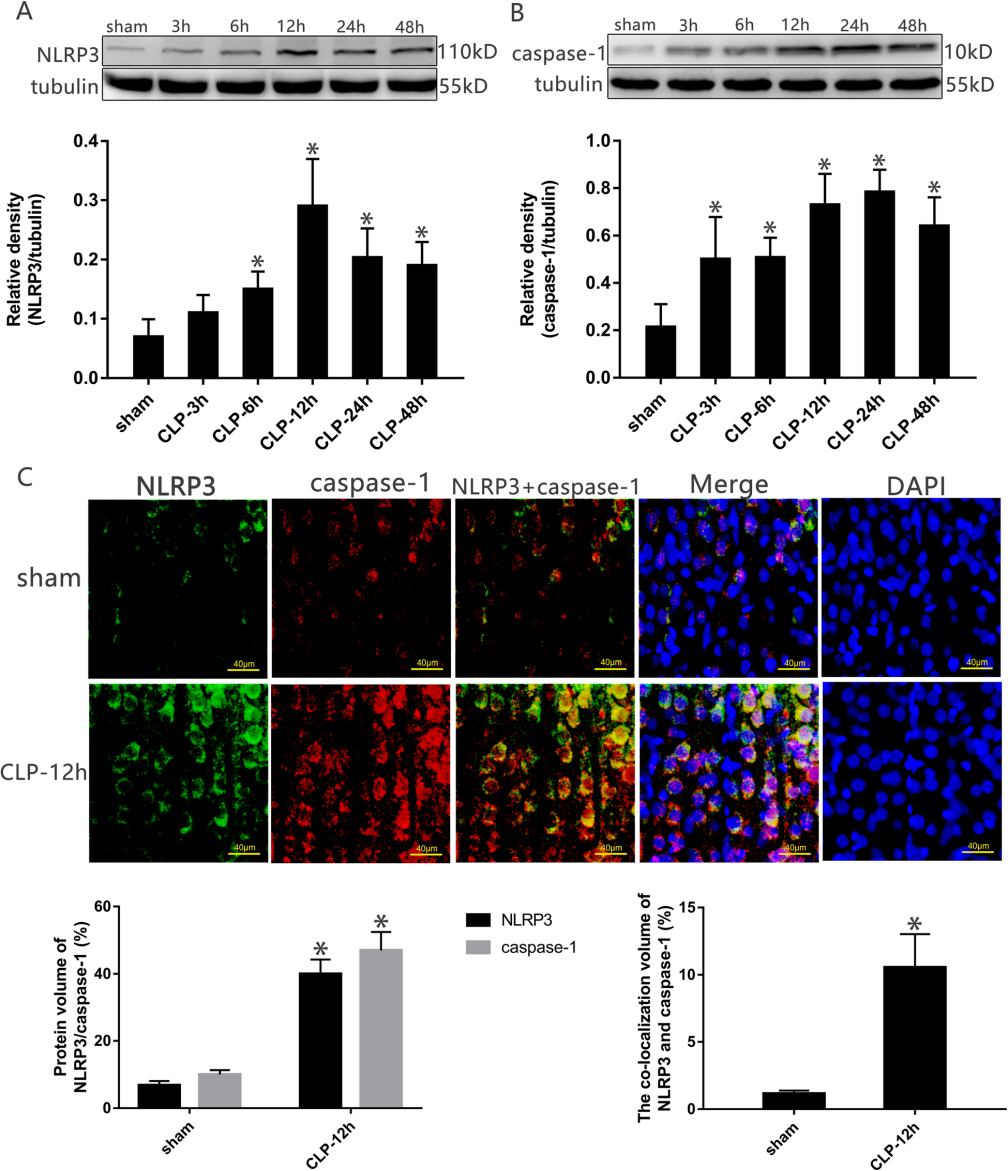
The expression of NLRP3 and caspase-1 in each group at different time points. **a, b** NLRP3 and caspase-1 in the cortex harvested at specific time points after CLP. The protein levels were normalized to those of tubulin protein and are shown as relative arbitrary units. **c** Fluorescence of NLRP3 and caspase-1 in the cortex at specific time points after CLP. Three-color staining for anti-NLRP3 antibody (green), anti-caspase-1 (red), and nucleus (blue), the protein volume and coincidence rate are represented by histograms. Scale bar = 40 μm. Values are expressed as mean ± SEM (*n* = 6 in each group; *P < 0.05 vs. sham group, in the corresponding group)

Cortical tissue was harvested from the rats at specific time points. Western blotting showed that compared with the sham group, the expression levels of NLRP3 and caspase-1 were significantly increased at 6, 12, 24, and 48 h (*P* < 0.05, Fig. 4a, b). Immunofluorescence also showed that NLRP3 and caspase-1 signals were significantly enhanced compared with in the sham operation group. The coincidence ratio of the two proteins was analyzed by fluorescence co-location, and CLP rats showed significantly higher co-localization of these proteins than the sham group (*P* < 0.05, Fig. 4c).

### Increase in inflammatory cytokines in the cortex after CLP surgery

**Fig. 5:**
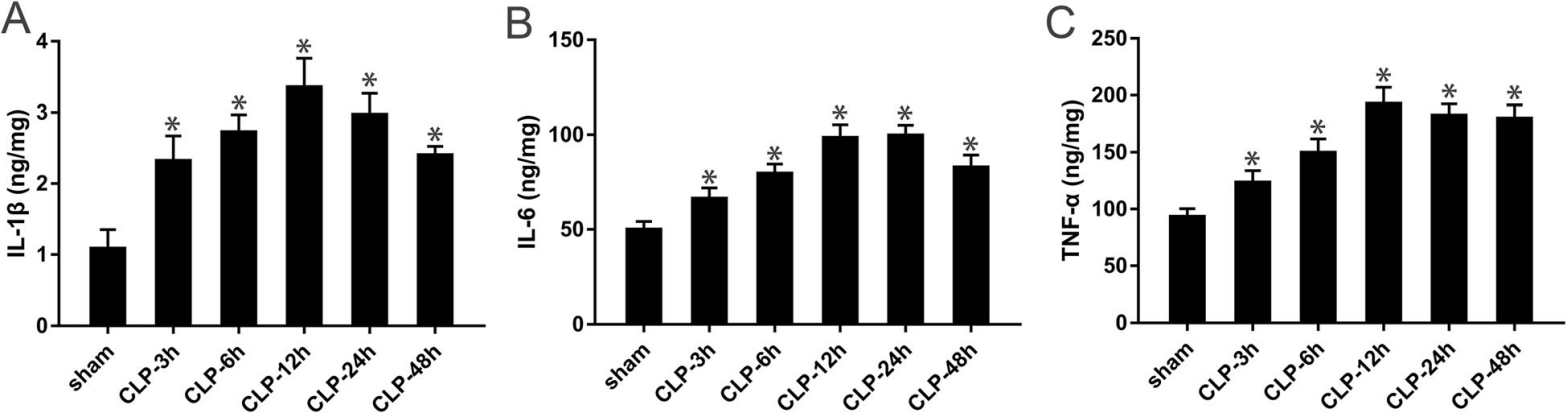
The levels of IL-1β, IL-6, and TNF-α in the cortical supernatant at specific time points. **a** IL-1β; **b** IL-6; **c** TNF-α. Data is represented by histograms. Values are expressed as mean ± SEM (*n* = 6 in each group. *P < 0.05 vs. sham group)

After the cortical supernatant was collected at specific time points, the relevant inflammatory factors were detected by ELISA kits. Compared with the sham group, the CLP group showed significantly higher levels of inflammatory factors in the cortex at each time point, with the levels of IL-1β, IL-6, and TNF-α peaking at 12 h (*P* < 0.05, Fig. 5a–c).

### Changes in p38 MAPK and ERK signaling pathways in the cortex

**Fig. 6:**
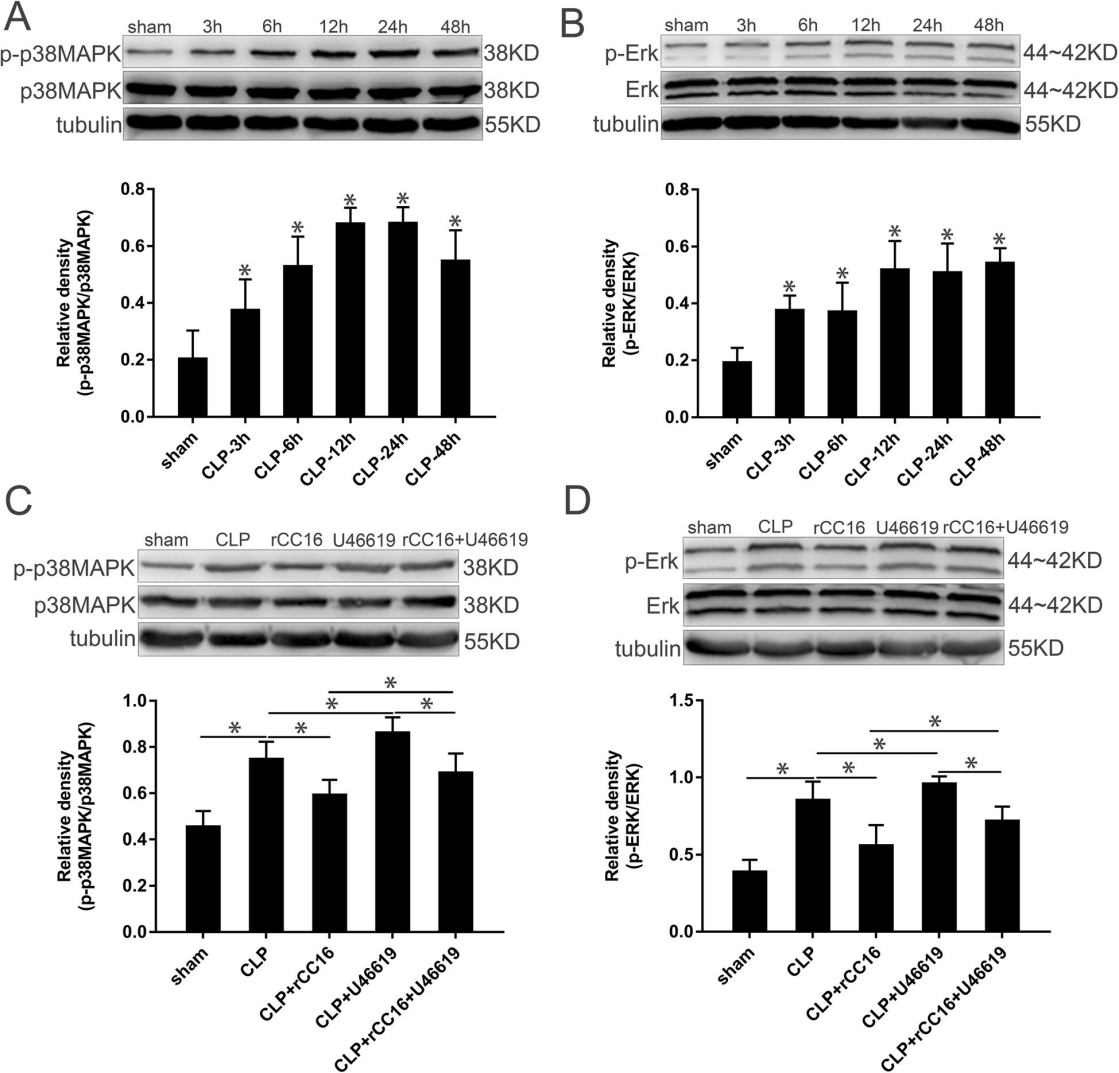
p-p38 MAPK, p38 MAPK, p-ERK, and ERK levels in each group. **a** p-p38 MAPK and p38 MAPK in the cortex harvested at specific time points after CLP. **b** p-ERK and ERK in the cortex harvested at specific time points after CLP. **c** p-p38 MAPK and p38 MAPK in the cortex harvested at 12 h. **d** p-ERK and ERK in the cortex harvested at 12 h. The protein levels were normalized to those of tubulin protein and are shown as relative arbitrary units. Data is represented by histograms. Values are expressed as mean ± SEM (*n* = 6 in each group; *P < 0.05)

The cortical tissue of the rats was collected at specific time points and the protein expression in the tissue was detected by western blot. Compared with the sham group, the expression of phosphorylated p38 MAPK (p-p38 MAPK) in the CLP group increased significantly over time after CLP, peaking at 12 and 24 h and then decreasing after 48 h (*P* < 0.05, Fig. 6a). In the CLP group, the phosphorylation of ERK was also significantly increased, reaching a maximum at 12 h and remaining high thereafter (*P* < 0.05, Fig. 6b). To explore the changes in the two signaling pathways in the cortex after drug administration, we chose the time point of 12 h for comparison. Compared with the CLP group, phosphorylation of p38 MAPK and ERK decreased in the CLP + rCC16 group and increased in the CLP + U46619 group. The phosphorylation of p38 MAPK and ERK decreased in the CLP + rCC16 + U46619 group compared with in the CLP + rCC16 group. In addition, the phosphorylation of p38 MAPK and ERK in the CLP + rCC16 + U46619 group was lower than that in the CLP + U46619 group (*P* < 0.05, Fig. 6c and d).

### Changes in NLRP3 and caspase-1 expression in the cortex after drug administration

**Fig. 7:**
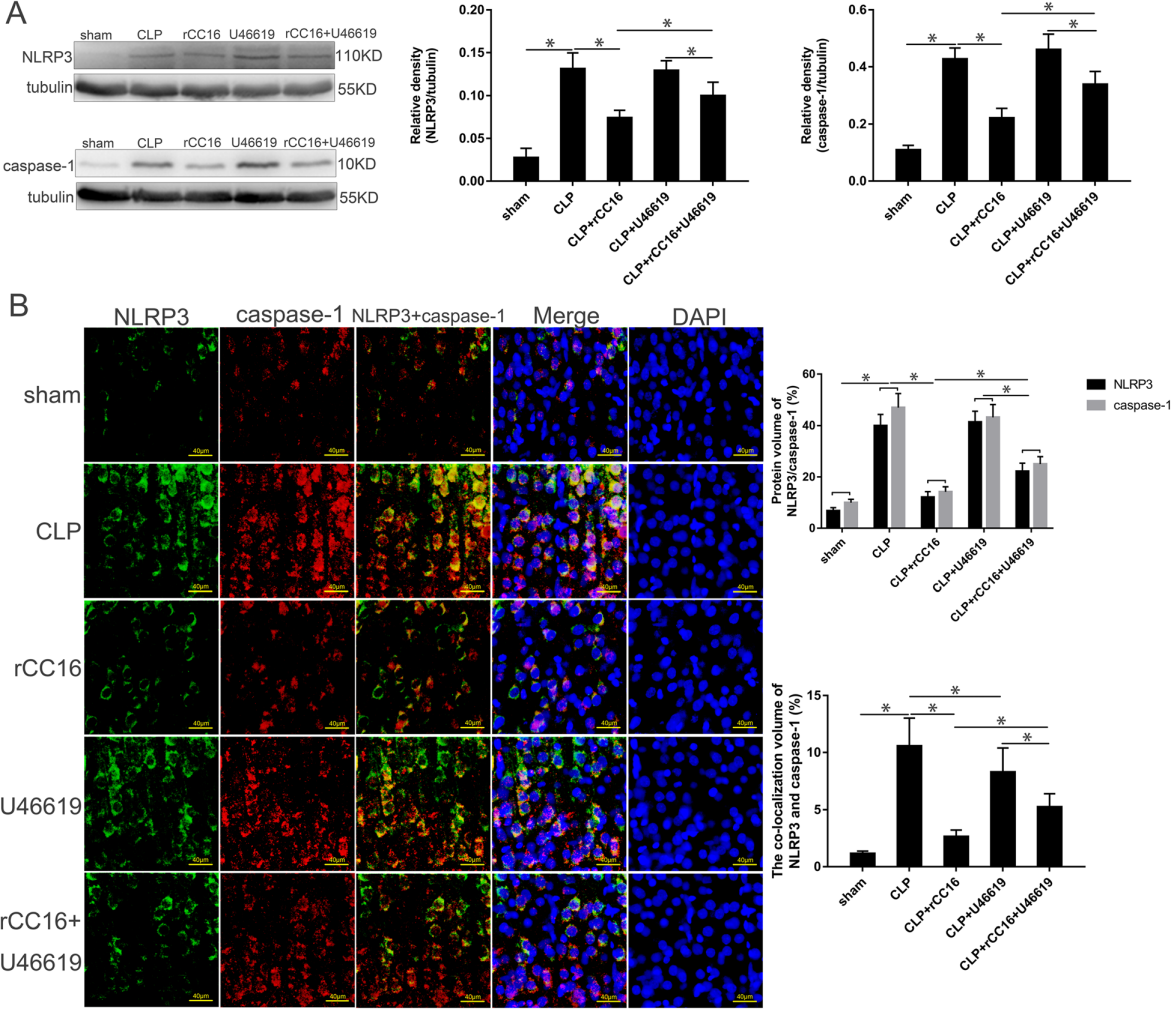
The expression of NLRP3 and caspase-1 in the cortex at 12 h. **a** NLRP3 and caspase-1 in the cortex harvested in different groups. The protein levels were normalized to those of tubulin protein and are shown as relative arbitrary units. **b** Fluorescence of NLRP3 and caspase-1 in the cortex in different groups. Three-color staining for anti-NLRP3 (green), anti-caspase-1 (red), and nucleus (blue), the protein volume and coincidence rate are represented by histograms. Scale bar = 40 μm. Values are expressed as mean ± SEM (*n* = 6 in each group; *P < 0.05)

To validate our findings, we chose the time point of 12 h for more detailed investigation and comparison. Western blotting showed that compared with the CLP group, the expression of NLRP3 and caspase-1 in the cortex of the CLP + rCC16 group was significantly decreased, while the CLP + U46619 group showed no significant change; after further administration of U46619 to the CLP + rCC16 group, NLRP3 and caspase-1 expression in the cortex of rats was significantly increased and protein expression in the CLP + rCC16 + U46619 group was lower than that in the CLP + U46619 group (*P* < 0.05, Fig. 7a). Analysis of the protein volume by measuring fluorescent signals showed similar results to those obtained by western blot analysis. The CLP group and CLP + U46619 group showed the highest protein volumes, with no significant difference between these two groups. The protein volume of NLRP3 and caspase-1 in the CLP + rCC16 + U46619 group was lower than that of the previous two groups, followed by the CLP + rCC16 group (*P* < 0.05, Fig. 7b). Further fluorescence co-localization showed that the protein coincidence rate was highest in the CLP group, with both the CLP + rCC16 group and CLP + U46619 group showing lower values than the CLP group. The protein coincidence rate in the CLP + U46619 group was higher than that in the CLP + rCC16 + U46619 group, while that of the CLP + rCC16 group was lower than that of the CLP + rCC16 + U46619 group (*P* < 0.05, Fig. 7b).

### Changes in inflammatory cytokines in the cortex after drug administration

**Fig. 8:**
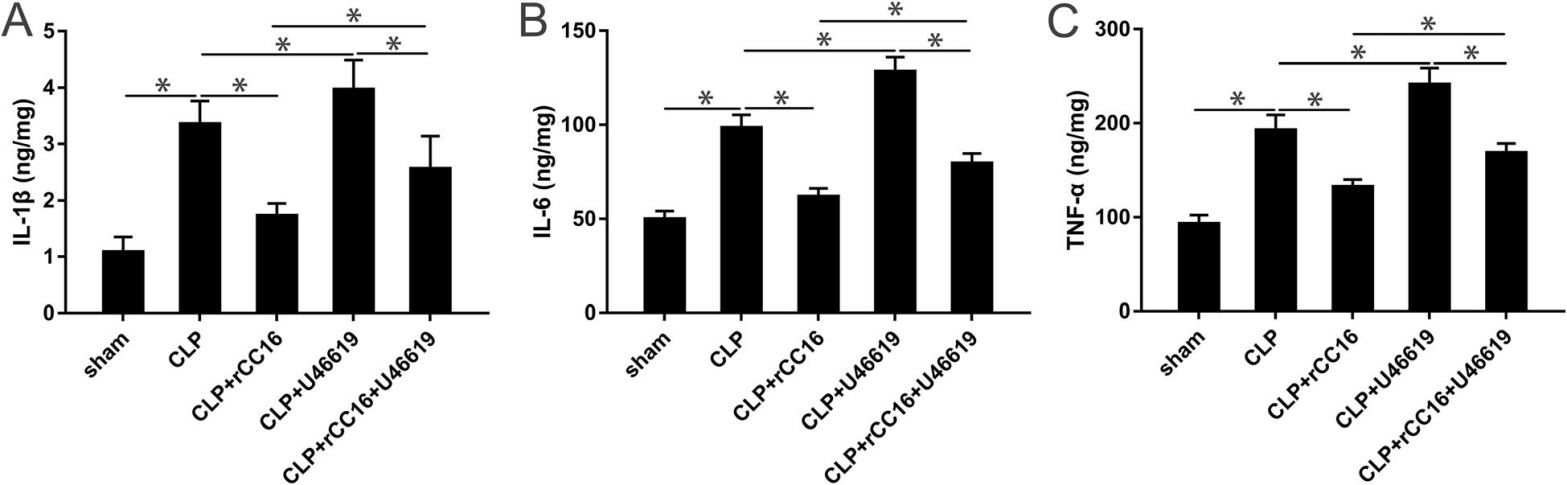
The levels of IL-1β, IL-6, and TNF-α in the cortical supernatant at 12 h. **a** The level of IL-1β harvested in different groups. **b** The level of IL-6 harvested in different groups. **c** The level of TNF-α harvested in different groups. Data is represented by histograms. Values are expressed as mean ± SEM (*n* = 6 in each group; *P < 0.05)

ELISA was again used to detect the relevant inflammatory factors in the cortical supernatant. After CLP, the level of inflammatory cytokines increased and reached a maximum value at 12 h. Therefore, the time point of 12 h was selected for subsequent analysis. Compared with the CLP group, there was a significant decrease in the levels of IL-1β, IL-6, and TNF-α in the cortical supernatant of the CLP + rCC16 group, whereas those in the CLP + U46619 group were increased. Compared with the CLP + rCC16 + U46619 group, the levels of IL-1β, IL-6, and TNF-α were lower in the CC16 + rCC16 group but higher in the CC16 + U46619 group (*P* < 0.05, Fig. 8a–c).

## Discussion

In this study, we observed that the survival percentage of rats in the CLP group was significantly lower than that in the sham group, with worse clinical performance and worsening vital signs (decreased MAP and increased HR), suggesting that establishment of the sepsis model using CLP was successful [27]. Compared with the sham group, the neurobehavioral scores of rats in the CLP group were significantly decreased. And acute traumatic changes also occurred in the cortical tissues, indicating that the sepsis could induce brain injury by CLP [24].

Pyroptosis is a recently discovered type of cell death. Although a certain degree of pyroptosis can confer a host with a defensive mechanism against infectious diseases, excessive pyroptosis is harmful [6]. First, NLRs, as intracellular pattern receptors, recognize invading pathogens and form NLR-containing inflammasomes, subsequently activating caspase-1 [28]. NLRP3 is a representative NLR. The active caspase-1 causes the plasma membrane pore to form difference between intracellular and extracellular ion. High intracellular osmotic pressure causes tissue fluid to enter the cell, leading to cell swelling and dissolution, and releasing of intracellular inflammatory factors [29–31]. These inflammatory factors include IL-1β, IL-6, and TNF-α [6, 32]. Therefore, by monitoring the changes in NLRP3 and caspase-1, we observed pyroptosis of cortical neurons in rats with sepsis-induced brain injury after different treatments.

Western blot analysis showed that compared with the sham group, the expression of NLRP3 and caspase-1 in the cortex of the CLP group rats was significantly increased. The same results were also obtained by immunofluorescence assay, with the fluorescence signals of the two proteins showing a very high coincidence rate in the neurons, indicating that the increase in NLRP3 and caspase-1 was the common factor in cell pyroptosis. This is consistent with the findings of Fu et al. [5], who found that in sepsis-associated encephalopathy, NLRP3 and caspase-1 induced neuronal pyroptosis in the hippocampus, resulting in cognitive impairment in mice. In addition, we found that compared with the sham group, the levels of inflammatory cytokines (IL-1β, IL-6, and TNF-α) in the cortex of rats in the CLP group were also significantly increased, with a peak time slightly later than that of NLRP3 because the release of inflammatory cytokines after cell lysis is a downstream result of NLRP3/caspase-1 dependent pore formation [6, 29, 33]. Based on the above results, we found that with the aggravation of pyroptosis at different time points and release of inflammatory factors, the survival percentage of the rats decreased; the clinical performance and vital signs deteriorated; and brain injury worsened.

To further study the mechanism of pyroptosis, we attempted to identify the signaling pathway regulating pyroptosis. It has been experimentally demonstrated that in acute lung injury, blocking of the p38 MAPK signaling pathway can inhibit the pyroptosis of macrophages and reduce the release of inflammatory factors [20]. Numerous experiments have confirmed that inhibition of MAPKs can inhibit the activation of NLRs and caspase-1 [34–37]. In this study, we also found that compared with the sham group, the phosphorylation of p38 MAPK and ERK in the cortex of rats in the CLP group increased significantly. Thus, activation of the p38 MAPK and ERK signaling pathways in sepsis-induced brain injury may be related to the activation of pyroptosis.

The rCC16 has been reported to have a protective effect on the development of neonates and can regulate the p38 MAPK signaling pathway to reduce the release of inflammatory factors [10, 19]. Therefore, we reasoned that rCC16 could reduce the pyroptosis of newborn rat cortical nerve cells by inhibiting MAPKs in sepsis. Because the fluctuations in the detection indices in CLP group rats were most obvious at 12 h, we selected this time point for subsequent analysis.

In the CLP + rCC16 group, the expression of phosphorylated p38 MAPK and ERK was reduced. This was accompanied by reduced expression of NLRP3 and caspase-1 and decreased release of related inflammatory factors (IL-1β, IL-6, and TNF-α). This indicates that the p38 MAPK and ERK signaling pathways, as well as pyroptosis, were inhibited, leading to the alleviation of inflammation. Compared with the CLP group, the survival percentage was better in the CLP + rCC16 group and the clinical performance, vital signs, neuroreflex, and cortical pathological changes were attenuated. Therefore, rCC16 can inhibit the p38 MAPK and ERK signaling pathways, inhibit pyroptosis, and alleviate the inflammatory response. However, it remained unclear whether rCC16 inhibited pyroptosis by inhibiting the p38 MAPK and ERK signaling pathways.

To verify whether rCC16 inhibits pyroptosis through the p38 MAPK and ERK signaling pathways, we further administered CLP rats with U46619, an agonist for p38 MAPK and ERK. Compared with the CLP + rCC16 group, the p38 MAPK and ERK signaling pathways were reactivated in the CLP + rCC16 + U46619 group, with more active pyroptosis and enhanced inflammation; additionally, the survival percentage decreased, and the clinical performance, vital signs, neuro reflex, and pathological changes worsened. After activating the p38 MAPK and ERK signaling pathways, U46619 likely activated pyroptosis, leading to a series of cascading changes that reversed the protective effect of rCC16 in rats. Based on these results, rCC16 can inhibit pyroptosis and reduce inflammation by blocking the p38 MAPK and ERK signaling pathways in newborn septic rats, thereby playing a protective role.

Compared with the CLP group, CLP rats administered U46619 alone showed a worse clinical performance and more severe cortical pathological changes. The p38 MAPK and ERK signaling pathways in this group were more effectively activated and inflammatory factors were released. Interestingly, however, the CLP + U46619 group showed no significant increase in the expression of NLRP3 and caspase-1, and fluorescence co-localization suggested a decrease their coincidence. This may be due to the fact that not only caspase-1 is a driving protein for pyroptosis, but more caspases are also activated proteins for pyroptosis [38–40]. When the MAPK signaling pathways are overactivated, the expression of caspases (caspase-4/5/11) is increased [41–44], which may affect caspase-1-dependent pyroptosis. However, the reason for this phenomenon requires further study.

There were some limitations to this study. The exact mechanism causing rCC16 to block the p38 MAPK and ERK signaling pathways remains unknown, warranting further investigations. In addition, although rCC16 stabilized the vital signs of newborn rats with sepsis, resuscitated the nerve reflex, and alleviated the pathological changes of the cortex, this study could not prove that these protective effects were directly related to the inhibition of pyroptosis. Finally, this study only explored caspase-1-dependent pyroptosis, but we still need further study to elucidate the mechanism of other caspases (caspase-4/5/11) activating pyroptosis in sepsis.

## Conclusions

In summary, in neonatal sepsis, the cortical p38 MAPK and ERK signaling pathways are activated, resulting in pyroptosis and inflammation. rCC16 protects against pyroptosis and inflammation by blocking these signaling pathways. Although the mechanism still need be further clarified, we provide preliminary evidence that rCC16 can play a role in the prevention and treatment of sepsis. These results provide a new direction for the clinical treatment of sepsis.

## Data Availability

The datasets used and/or analyzed during the current study are available from the corresponding author on reasonable request.

## Abbreviations

CLP: Cecal ligation and perforation
rCC16: Recombinant club cell protein
NLRP3: Nucleotide-binding domain leucine-rich repeat-containing pyrin domain-containing 3
MAPK: Mitogen-activated protein kinase
ERK: Extracellular signal-related kinase
TNF-α: Tumor necrosis factor alpha
IL: Interleukin
CC16: Club cell protein
MAP: Mean arterial pressure
HR: Heart rate
PFA: Paraformaldehyde
ECL: Enhanced chemiluminescence
PBS: Phosphate-buffered saline
DAPI: 4, 6-Diamidino-2-phenylindole
SPSS: Statistical Package for Social Sciences

## References

[1] Ning Q, Liu Z, Wang X, Zhang R, Zhang J, Yang M (2017). Neurodegenerative changes and neuroapoptosis induced by systemic lipopolysaccharide administration are reversed by dexmedetomidine treatment in mice. Neurol Res..

[2] Taccone FS, Scolletta S, Franchi F, Donadello K, Oddo M (2013). Brain perfusion in sepsis. Curr Vasc Pharmac..

[3] Zhang LN, Wang XT, Ai YH, Guo QL, Huang L, Liu ZY (2012). Epidemiological features and risk factors of sepsis-associated encephalopathy in intensive care unit patients: 2008-2011. Chin Med J (Engl)..

[4] Cotena S, Piazza O (2012). Sepsis-associated encephalopathy. J Transl Med..

[5] Fu Q, Wu J, Zhou XY, Ji MH, Mao QH, Li Q, et al. NLRP3/Caspase-1 pathway-induced pyroptosis mediated cognitive deficits in a mouse model of sepsis-associated encephalopathy. Inflammation. 2019;42(1):306–18. 10.1007/s10753-018-0894-4 PMC6394578 30276509

[6] Bergsbaken T, Fink SL, Cookson BT (2009). Pyroptosis: host cell death and inflammation. Nat Rev Microbiol..

[7] Pétrilli V, Papin S, Dostert C, Mayor A, Martinon F, Tschopp J (2007). Activation of the NALP3 inflammasome is triggered by low intracellular potassium concentration. Cell Death Differ..

[8] Zhou RX, Qu Y, Huang Q, Sun XM, Mu DZ, Li XH (2019). Recombinant CC16 regulates inflammation, oxidative stress, apoptosis and autophagy via the inhibition of the p38MAPK signaling pathway in the brain of neonatal rats with sepsis. Brain Res..

[9] Hagman C, Björklund LJ, Hellgren G, Tufvesson E, Hansen-Pupp I (2018). Club cell secretory protein (CC16) in gastric fluid at birth and subsequent lung disease in preterm infants. Pediatr Pulmonol..

[10] Levine CR, Gewolb IH, Allen K, Welch RW, Melby JM, Pollack S (2005). The safety, pharmacokinetics, and anti-inflammatory effects of intratracheal recombinant human Clara cell protein in premature infants with respiratory distress syndrome. Pediatr Res..

[11] Shashikant BN, Miller TL, Welch RW, Pilon AL, Shaffer TH, Wolfson MR (2005). Dose response to rhCC10-augmented surfactant therapy in a lamb model of infant respiratory distress syndrome: physiological, inflammatory, and kinetic profiles. J Appl Physiol..

[12] Chandra S, Davis JM, Drexler S, Kowalewska J, Chester D, Koo HC (2003). Safety and efficacy of intratracheal recombinant human Clara cell protein in a newborn piglet model of acute lung injury. Pediatr Res..

[13] Chen Y, Ba L, Huang W, Liu Y, Pan H, Mingyao E (2017). Role of carvacrol in cardioprotection against myocardial ischemia/reperfusion injury in rats through activation of MAPK/ERK and Akt/eNOS signaling pathways. Eur J Pharmacol..

[14] Cheng W, Zhao Q, Xi Y, Li C, Xu Y, Wang L (2015). IFN-β inhibits T cells accumulation in the central nervous system by reducing the expression and activity of chemokines in experimental autoimmune encephalomyelitis. Mol Immunol..

[15] Kim H, Moore SA, Johnston MG (2014). Potential for intranasal drug delivery to alter cerebrospinal fluid outflow via the nasal turbinate lymphatics. Fluids Barriers CNS..

[16] Yun DH, Song HY, Lee MJ, Kim MR, Kim MY, Lee JS (2009). Thromboxane A2 modulates migration, proliferation, and differentiation of adipose tissue-derived mesenchymal stem cells. Exp Mol Med.

[17] Kim JM, Koo YK, Jin J, Lee YY, Park S, Yun-Choi HS (2009). Augmentation of U46619 induced human platelet aggregation by aspirin. Platelets..

[18] Hamilton JR, Cornelissen I, Coughlin SR (2004). Impaired hemostasis and protection against thrombosis in protease-activated receptor 4-deficient mice is due to lack of thrombin signaling in platelets. J Thromb Haemost..

[19] Li DD, Ren WY, Jiang ZL, Zhu L (2018). Regulation of the NLRP3 inflammasome and macrophage pyroptosis by the p38 MAPK signaling pathway in a mouse model of acute lung injury. Mol Med Rep..

[20] Pang M, Yuan YY, Wang D, Li T, Wang D, Shi XH (2017). Recombinant CC16 protein inhibits the production of pro-inflammatory cytokines via NF-κB and p38 MAPK pathways in LPS-activated RAW264.7 macrophages. Acta Biochim Biophys Sin (Shanghai).

[21] Daniel R, Markus SH, Michael AF, Peter AW (2009). Immunodesign of experimental sepsis by cecal ligation and puncture. Nat Protoc..

[22] Hennenberg M, Acevedo A, Wiemer N, Kan A, Tamalunas A, Wang Y (2017). Non-adrenergic, tamsulosin-insensitive smooth muscle contraction is sufficient to replace α1-adrenergic tension in the human prostate. Prostate..

[23] Qin X, Hurn PD, Littleton-Kearney MT (2005). Estrogen restores postischemic sensitivity to the thromboxane mimetic U46619 in rat pial artery. J Cereb Blood Flow Metab..

[24] Kafa IM, Uysal M, Bakirci S, AybERK KM (2010). Sepsis induces apoptotic cell death in different regions of the brain in a rat model of sepsis. Acta Neurobiol Exp (Wars)..

[25] Abdel-Rahman A, Shetty AK, Abou-Donia MB (2002). Acute exposure to sarin increases blood brain barrier permeability and induces neuropathological changes in the rat brain: dose-response relationships. Neuroscience..

[26] Ni H, Rui Q, Lin X, Li D, Liu H, Chen G (2019). 2-BFI provides neuroprotection against inflammation and necroptosis in a rat model of traumatic brain injury. Front Neurosci.

[27] Alejandra GG, Luiz FPDF, Maurício RES (2004). Experimental models of sepsis and septic shock: an overview. Acta Cir Bras.

[28] Fernandes AT, Wu J, Yu JW, Datta P, Miller B, Jankowski W (2007). The pyroptosome: a supramolecular assembly of ASC dimers mediating inflammatory cell death via caspase-1 activation. Cell Death Differ..

[29] Fink SL, Cookson BT (2006). Caspase-1-dependent pore formation during pyroptosis leads to osmotic lysis of infected host macrophages. Cell Microbiol..

[30] Kawai T, Akira S (2007). TLR signaling. Semin. Immunol..

[31] Kufer TA, Sansonetti PJ (2007). Sensing of bacteria: NOD a lonely job. Curr Opin Microbiol..

[32] Miggin SM, Pålsson-McDermott E, Dunne A, Jefferies C, Pinteaux E, Banahan K (2007). NF-kappaB activation by the Toll-IL-1 receptor domain protein MyD88 adapter-like is regulated by caspase-1. Proc Natl Acad Sci USA..

[33] Fantuzzi G, Dinarello CA (1999). Interleukin-18 and interleukin-1 beta: two cytokine substrates for ICE (caspase-1). J Clin Immunol..

[34] Su WJ, Zhang Y, Chen Y, Gong H, Lian YJ, Peng W (2017). NLRP3 gene knockout blocks NF-κB and MAPK signaling pathway in CUMS-induced depression mouse model. Behav Brain Res.

[35] Fann DY, Lim YA, Cheng YL, Lok KZ, Chunduri P, Baik SH (2018). Evidence that NF-κB and MAPK signaling promotes NLRP inflammasome activation in neurons following ischemic stroke. Mol Neurobiol..

[36] Choe JY, Jung HY, Park KY, Kim SK (2014). Enhanced p62 expression through impaired proteasomal degradation is involved in caspase-1 activation in monosodium urate crystal-induced interleukin-1β expression. Rheumatology (Oxford)..

[37] Philip NH, Dillon CP, Snyder AG, Fitzgerald P, Wynosky-Dolfi MA, Zwack EE (2014). Caspase-8 mediates caspase-1 processing and innate immune defense in response to bacterial blockade of NF-κB and MAPK signaling. Proc Natl Acad Sci USA..

[38] Galluzzi L, Vitale I, Aaronson SA, Abrams JM, Adam D, Agostinis P (2018). Molecular mechanisms of cell death: recommendations of the Nomenclature Committee on Cell Death 2018. Cell Death & Differentiation..

[39] Aachoui Y, Sagulenko V, Miao EA, Stacey KJ (2013). Inflammasome-mediated pyroptotic and apoptotic cell death, and defense against infection. Current Opinion in Microbiology..

[40] Vanden BT, Demon D, Bogaert P, Vandendriessche B, Goethals A, Depuydt B (2014). Simultaneous targeting of IL-1 and IL-18 is required for protection against inflammatory and septic shock. Am J Respir Crit Care Med..

[41] Napier BA, Brubaker SW, Sweeney TE, Monette P, Rothmeier GH, Gertsvolf NA (2016). Complement pathway amplifies caspase-11-dependent cell death and endotoxin-induced sepsis severity. J Exp Med..

[42] Wang M, Tsai BM, Turrentine MW, Mahomed Y, Brown JW, Meldrum DR (2005). p38 mitogen activated protein kinase mediates both death signaling and functional depression in the heart. Ann Thorac Surg..

[43] Wang M, Tsai BM, Kher A, Baker LB, Wairiuko GM, Meldrum DR (2005). Role of endogenous testosterone in myocardial proinflammatory and proapoptotic signaling after acute ischemia-reperfusion. Am J Physiol Heart Circ Physiol..

[44] Hur J, Kim SY, Kim H, Cha S, Lee MS, Suk K (2001). Induction of caspase-11 by inflammatory stimuli in rat astrocytes: lipopolysaccharide induction through p38 mitogen-activated protein kinase pathway. FEBS Lett..

